# An induced pluripotent stem cell model of Schwann cell differentiation reveals NF2- related gene regulatory networks

**DOI:** 10.21203/rs.3.rs-6775534/v1

**Published:** 2025-06-16

**Authors:** Olivia Lazaro, Sihong Li, William Carter, Jake Smiley, Oluwamayowa Awosika, Sylvia Robertson, Angela Haskell, Raven Hinkel, Brooke E. Hickey, Steven P. Angus, Austin House, D. Wade Clapp, Abdul Q. Syed, Travis S. Johnson, Steven D. Rhodes

**Affiliations:** Indiana Biosciences Research Institute; Indiana Biosciences Research Institute; Indiana Biosciences Research Institute; Indiana Biosciences Research Institute; Indiana Biosciences Research Institute; Indiana Biosciences Research Institute; Indiana Biosciences Research Institute; Indiana Biosciences Research Institute; Indiana University School of Medicine; Indiana University School of Medicine; Indiana Biosciences Research Institute; Indiana University School of Medicine; Indiana Biosciences Research Institute; Indiana Biosciences Research Institute; Indiana University School of Medicine

**Keywords:** NF2, Schwannoma, hiPSC, scRNA-seq, gene co-expression analysis, systems biology

## Abstract

Schwann cells are vital to development and maintenance of the peripheral nervous system and their dysfunction has been implicated in a range of neurological and neoplastic disorders, including *NF2*-related schwannomatosis (*NF2*-SWN). We have developed a novel human induced pluripotent stem cell (hiPSC) model for the study of Schwann cell differentiation in health and disease. We performed transcriptomic, immunofluorescence, and morphological analysis of hiPSC derived Schwann cell precursors (SPCs) and terminally differentiated Schwann cells (SCs) representing distinct stages of development. To further validate our findings, we performed integrated, cross-species analyses across multiple external datasets at bulk and single cell resolution. Our hiPSC model of Schwann cell development shared overlapping gene expression signatures with human amniotic mesenchymal stem cell (hAMSCs) derived SCs and *in vivo* mouse models, but also revealed unique features that may reflect species-specific aspects of Schwann cell biology. Moreover, we have identified gene co-expression modules that are dynamically regulated during hiPSC to SC differentiation associated with ear and neural development, cell fate determination, the *NF2* gene, and extracellular matrix (ECM) organization. Through integrated analysis of multiple datasets and genetic disruption of NF2 via CRISPR-Cas9 gene editing in hiPSC derived SCPs, we have identified a series of novel ECM associated genes regulated by Merlin. Our hiPSC model further provides a tractable platform for studying Schwann cell development in the context of rare diseases such as *NF2*-SWN which lack effective medical therapies.

## INTRODUCTION

Schwann cells (SCs) are glial cells that originate in the neural crest and play a crucial role in myelinating peripheral nerves [1]. Myelination enhances the speed and efficiency of nerve conduction by forming a single spiraling myelin sheath around an axon [2]. Non-myelinating SCs also provide unmyelinated axons with trophic support and mechanical cushioning [3]. Deregulated SC development can give rise to tumors of the peripheral and cranial nerves called schwannomas [4]. Schwannomas have unpredictable clinical behavior, significant morbidities, and exceedingly limited therapeutic options available for their treatment [5].

Most vestibular schwannomas occur unilaterally; however, approximately 10% of cases involve both sides and are frequently associated with *NF2*-SWN [6]. Schwannoma development is often associated with an autosomal dominant, heritable genetic condition called *NF2*-related schwannomatosis (*NF2*-SWN, formerly known as neurofibromatosis type 2) [7], which is caused by pathogenic variants in the *NF2* tumor suppressor gene, which encodes Merlin, a cytoskeletal adaptor protein with diverse biochemical functions [8]. The development of bilateral vestibular schwannomas is a hallmark of *NF2*-SWN [9]. These tumors involve the eight cranial nerves, which are critical for hearing and balance. Affected individuals often suffer significant morbidities including tinnitus, sensorineural hearing loss, and impairment of balance and coordination [7].

To better understand the molecular mechanisms underlying schwannoma genesis in *NF2*-SWN, the development of new model systems for normal and diseased SCs is imperative. Human induced pluripotent stem cells (hiPSCs) have demonstrated promise in modeling a multitude of neurological diseases and cell types, including but not limited to hiPSC-derived astrocytes [10–13], microglia [14–16], and neurons [17, 18]. Direct conversion of human fibroblasts to hiPSC-deviled neurons has also been achieved [18]. These iPSC-derived cells have been employed to interrogate neuropsychiatric diseases [19–21], neurodegenerative diseases [14, 21, 22], and gliomas [23–25]. However, beyond gliomas which occur in the central nervous system, considerably less progress has been made in the field of iPSC modeling of peripheral nervous system derived tumors including schwannoma [26].

To address this unmet need, we have developed a novel hiPSC SC model that can be used to study SC differentiation in health and disease. We began by characterizing a novel hiPSC line for pluripotency markers and embryoid body formation and then proceeded to perform transcriptomic profiling of hiPSCs at multiple stages of SC differentiation to unveil broad networks of differentially expressed genes (DEGs) associated with SC lineage maturation, which we compared to other previously published datasets (Table 1). To further characterize complex gene regulatory networks (GRNs) associated with SC differentiation, we employed a systems biology approach, utilizing weighted gene co-expression network analysis (WGCNA) to identify modules of transcriptionally associated genes [27] and study their temporal dynamics. The putative roles of these modules were further defined using functional enrichment analysis [28]. To validate our findings, we compared the DEGs and gene modules derived from our hiPSC induced SCs with two independent single cell and bulk transcriptomic datasets of human SC differentiation [29] and mouse sciatic nerve development [30]. Then our consistent top DEGs were evaluated in a disparate dataset of NF2 KO iPSC-derived SCs to evaluate their association to NF2.

## METHODS

### hiPSC generation

hiPSC lines were generated from neonatal human dermal fibroblasts (HDFn) from Invitrogen (Catalog #: C-004–5C) using an Epi5^™^ Episomal iPSC Reprogramming Kit (ThermoFisher Scientific) according to manufacturer’s protocol. Transduced cells were plated on growth factor reduced Matrigel (Corning, 356231) coated culture dishes in the presence of ROCK inhibitor and maintained in Essential 8 (E8) medium. hiPSC colonies were mechanically isolated and expanded until at least passage 12 before differentiation. hiPSCs were characterized by staining with pluripotency markers and embryoid body (EB) formation. Mycoplasma testing was performed prior to SC differentiation.

### Schwann cell differentiation from hiPSCs

The hiPSC line IBRI.101.J was differentiated into SCs using a six-stage protocol over 31 days as described previously [31]. To start differentiation, IBRi 101.J hiPSC line was seeded on matrigel-coated culture plate in E8 media. When cell reached 50–60% confluence, the culture medium was replaced with Schwann cell basal 1 (SCB1) media (NDM containing 1x N2, 1x B27, 0.005% BSA, 2 mM GlutaMAX, 0.11 mM b-mercaptoethanol in advanced DMEM/F12 and Neurobasal medium (1:1 mix)) with 3 μM CHIR and 20 μM SB431542 for 6 days. On day 7, cells were fed with SCB1 media with 3 μM CHIR, 20 μM SB431542 and 50ng/mL NRG1 through day 24. SCB1 media was replaced with fresh medium daily, and the cells were routinely dissociated with Accutase and expanded once they reached 80% confluence. On day 25, media was switched to Schwann cell basal 2 (SCB2) media (DMEM, 1x NEAA, 1x BME and 2% FBS) with 200 ng/mL NRG1, 4 ng/mL Forskolin, 100nM RA-all Trans and 10ng/mL PDGF-BB for 3 days, then to SCB2 media with 200 ng/mL NRG1 and 10 ng/mL PDGF-BB for 2 days, and finally to SCB2 media with 200 ng/mL NRG1 for 2 more days. By day 31, terminal differentiation to SC was completed. Samples were collected at days 0, 3, 6, 12, 18, 24 and at a uniform 70% confluence in all cases for bulk RNA sequencing. Cell pellets were collected and flash frozen at −80°C from three wells for subsequent RNA extraction and RNA-seq, while one well was retained for continued passaging to the next stage.

### Immunoflourescence staining of hiPSC-derived Schwann cell precursors and SCs

We used immunofluorescence (IF) analysis to validate pluripotency markers, three germ layer embryoid body formation and SC marker genes in hiPSCs, Schwann precursor cells (SCPs) and SCs [32]. For immunostaining, cells were fixed with 4% Paraformaldehyde for 10 min. The fixed cells were washed with PBS three times and then blocked and permeabilized with 0.3% Triton X-100 (Sigma) in 1% Bovine Serum Albumin (Life Technologies) and 10% FBS for 1 hr at room temperature. Cells were then incubated with primary antibodies 1:200 dilution of SOX2 (Abcam #AB274516), SSEA4 (Abcam #AB16287), NANOG (Cell signaling #4903), TRA-1–60 (Cell signaling #47465), and OCT4 (Santa Cruz #sc-5279) for pluripotency markers, 1:400 dilution of Vimentin (Cell signaling #5741S), 1:400 dilution of SOX17 (R&D #AF1924) and 1:500 dilution of βIII-tubulin (Promega #G7121) for mesoderm, endoderm and ectoderm staining, 1:200 dilution of S100β (Abcam #AB52642), 1:50 dilution of p75 (Abcam #AB3125), 1:100 dilution of HOXb7 (Santa Cruz #sc-81292), and 1:500 dilution of GAP43 (Abcam #AB12274) for SCPs and SCs staining at 4°C overnight, then with conjugated secondary antibodies with 1:500 dilution for 1 hr at room temperature. The coverslips were mounted on slides with DAPI to stain the nuclei (VectorLabs). Images were captured with an LSM 780 confocal microscope running Zen Black software. Immunocytochemistry analysis confirmed the expression of S100β, p75, HOXb7, and GAP43.

### Flow cytometry

The hiPSC derived SCPs and SCs were collected from culture plates using Accutase, resuspended and washed with PBS. The cells were then fixed with 4% formaldehyde in PBS for 10 min and washed three times with PBS. The fixed cells were blocked and permeabilized with 0.1% Triton X-100, 10% FBS, and 1% BSA in PBS for 1 hour. Subsequently, cells were incubated with isotype control rabbit IgG or primary antibody for SOX10 (Abcam #AB155279), GAP43 (Abcam #AB12274), S100β (Abcam #AB52642) overnight at 4°C. After the antibody reaction, cells were washed three times with PBS and incubated with fluorophore-conjugated secondary antibody for 1 hr at room temperature. Finally, cells were washed and resuspended in 500 μL of PBS and run with CytekAurora (Cytek Biosciences) and analyzed using FlowJo version 10.10.0 (Treestar Inc).

### RNA extraction, library preparation and sequencing

RNA was extracted using phenol-chloroform extraction with TRIzol LS Reagent (Life Technologies Corporation, #10296028). 1 mL of TRIzol LS Reagent was added to flash frozen cell pellets followed by 0.2 mL of chloroform. Tubes were shaken by hand for 15 seconds and incubated at room temperature for 2–3 minutes. Centrifugation was performed on samples at 4°C for 15 minutes at 13,200 rpm. The aqueous phase of samples was transferred to an RNase-free tube and an equal amount of 70% ethanol was added to each sample. Samples were vortexed and binding and washing of RNA was carried out according to the instructions of Invitrogen’s PureLink^™^ RNA Micro Scale Kit #12183016. Samples were eluted in 20 μL RNase-free water. The quality and quantity of RNA was determined using NanoDrop^™^ Spectrophotometer (Thermo Scientific).

RNA samples were shipped overnight on dry ice to Novogene for quality control (QC), library preparation and RNA-sequencing (RNA-seq) as further described below. RNA-seq was performed on a total of 21 samples, 3 replicates for each of the 7 timepoints. Consensus Assessment of Sequence And Variation (CASAVA) base recognition software was used from the Illumina Genome Analyzer.

### Initial processing of RNA-sequencing data

Raw reads were removed if they contained adapter sequences, greater than 10% undetermined bases, or more than 50% low-quality bases (quality scores less than 5). Sequenced reads were then aligned to the GRCh38 Genome using HISAT2 v2.0.5 and quantified with feature counts v1.5.0-p3. Fragments per kilobase of transcript per million mapped reads (FPKM) values were calculated from the read counts and coding sequence (CDS) lengths for each gene across all timepoints and replicates. The dataset we generated is denoted Schwann cell Indiana University (SC-IU) throughout our study.

### Data Cleaning and QC

Statistical analysis was performed using R (v4.1.1.). Genes with the lowest 10% mean FPKM across all timepoints were removed. Ensembl IDs for the filtered genes were converted to HGNC gene symbols using biomaRt (v2.52.0) and the Ensembl gene IDs from the Genome assembly GRCh38.p14 and NCBI RefSeq for annotation. The highest average FPKM Ensembl ID was used for HGNC symbols with multiple corresponding Ensembl IDs.

### Differential Gene Expression analysis

Differential gene expression analysis was performed with edgeR v3.22.5. The log_2_ fold change (Log_2_FC) was calculated between groups and the p-values (P) were corrected using the Benjamini & Hochberg method (BH) to account for the false discovery rate (FDR). Each timepoint (day 3, 6, 12, 18, 24, 31) was compared to baseline (day 0), resulting in 6 pairwise comparisons. DEGs with Log_2_FC > 5.0 and FDR < 0.001 were identified for each comparison. Functional enrichment analysis was performed on DEGs from each comparison using clusterProfiler v3.8.1 to test the statistical enrichment for genes within the Reactome, DO (Disease Ontology), and DisGeNET pathways. Terms with FDR < 0.05 were considered enriched. Functional enrichment on other gene sets derived from this analysis was performed using ToppGene [33].

### GRN Construction and Module Detection

Genes with a maximum expression below the median across all samples were removed to significantly reduce the number of uninformative genes that may bias the analysis. Weighted gene coexpression network analysis (WGCNA) module generation was conducted with an unsigned Pearson correlation coefficient (PCC) matrix. Soft thresholding power was calculated to reduce noise for gene correlations and emphasize correlations between genes per WGCNA documentation. A block size of 22000 was used for network construction and module detection as described previously [27]. Eigengenes were subsequently calculated for each module detected by WGCNA. Gene modules whose eigengene values changed significantly (P < 0.05) for at least one timepoint were identified by analysis of variance (ANOVA). Enrichment analysis was performed using clusterProfiler to identify significantly associated ontology terms for each module. All ontologies contained in clusterProfiler were evaluated, including BP (biological process), CC (cellular component), and MF (molecular function).

### Evaluating Schwann cell differentiation from amniotic mesenchymal stem cells via single cell RNA sequencing

Single cell RNA-seq (scRNA-seq) data of Schwann cell differentiation from Shantou University Medical College (SC-SUMC) was downloaded from Gene Expression Omnibus (GSE161066) [29]. The dataset contained both SCs and human amniotic mesenchymal stem cells (hAMSCs). The Seurat R package [34] was used for initial data cleaning and QC, removing cells with > 2,500 or < 200 unique feature counts as well as cells with > 5% mitochondrial RNAs. The hAMSC and SC samples were each normalized with SCTransform [35, 36] and then integrated with Harmony [37, 38]. Uniform manifold approximation and projection (UMAP) [39] was used as a final dimensionality reduction step. The integrated object was clustered, markers were identified for each cluster (|Log_2_FC|>0.58 and FDR < 0.05), and the relative cluster proportions were compared between hAMSCs and SCs. DGE analysis was also performed between hAMSCs and SCs to identify DEGs specific to either group (|Log_2_FC|>0.58 and FDR < 0.05).

### Evaluation of SC differentiation in mouse sciatic nerve

The Sciatic Nerve Atlas (SNAT) RNA-seq dataset FPKM values were downloaded from the SNAT data portal [30]. For gene names with more than one ID, the ID was selected with the highest average expression across all cells in the dataset. Genes with a transcripts per million (TPM) value greater than the median TPM value of all genes were retained for downstream analysis. Next, differential gene expression analysis was performed using the edgeR (3.34.1) package. Six comparisons were performed, one for each timepoint (E17.5, P1, P5, P14, P24, P60) compared to E13.5 as the reference timepoint. Genes were considered differentially expressed if they had an |Log_2_FC|>5 and FDR < 0.001. Genes were mapped to their appropriate human homolog using biomaRt (2.48.3) and the Dec 2021 Ensembl archive for both human and mouse.

### Evaluation of myelinating compared to non-myelinating mouse SCs

The SNAT myelination RNA-seq dataset FPKM values were downloaded from the SNAT data portal [30]. The initial processing and homolog mapping steps were as described above. DEGs were compared between myelinating SCs (mSCs) and non-myelinating SCs (nmSCs). Genes were considered differentially expressed if they had an |Log_2_FC|>5 and FDR < 0.001.

### Cellular subtyping of Schwann cells from mouse sciatic nerve

The integrated Seurat (4.3.0) object from the SNAT data portal (SS2_all) was downloaded [30] for analysis and comparison with additional datasets. UMAP was calculated on the integrated components using the first 10 dimensions. Marker genes were identified using the FindAllMarkers function. The homolog mapping steps were as previously described. In addition to identifying markers from the previously defined clusters, we also determined DEGs for various differentiation trajectories. Specifically, we identified DEGs using the FindMarkers function contrasting immature Schwann cells (iSCs) from mSCs, i.e., iSC vs. transition SC (tSC) and nm(R)SC, and DEGs contrasting iSCs from nmSCs, i.e., iSC vs. pmSC, mSC cluster 1, mSC cluster 2, and mSC cluster 3 (Table 1). Genes were considered differentially expressed if they had an |Log_2_FC|>0.58 and FDR < 0.05.

### Identification of conserved gene expression programs in differentiating SCs across myelination status and species

To examine the similarity of transcriptional programs between mouse and hiPSCs during SC differentiation, we compared DEGs from the SC-IU and SNAT RNA-seq datasets for each of the six time point comparisons. First, we calculated the Jaccard index (JI) for the up-regulated DEGs for each pairwise comparison between the SC-IU and SNAT datasets (i.e., E17 up-regulated DEGs (SNAT) and Day 3 up-regulated DEGs (SC-IU) JI, E17 up-regulated DEGs (SNAT) and Day 6 up-regulated DEGs (SC-IU) JI, etc.). The same process was conducted for the down-regulated DEGs, resulting in a total of 36 total JI values for the up-regulated and downregulated DEGs, respectively. We then averaged and normalized the JI values to obtain a square matrix with row and column sums of 1 to account for any skew toward either end of the differentiation process. We then repeated this process with less stringent DEG calling criteria (|Log_2_FC|>0.58 and FDR < 0.05). The consistency of the JI matrix was evaluated using a Student’s t-test of the diagonal of the matrix vs. the off-diagonal and a Spearman correlation coefficient (SCC) of the JI with the distance of each cell from the diagonal. Next, we compared the union of up-regulated and down-regulated DEGs from both the SC-IU and SNAT datasets to differentiation and developmental gene modules from the WGCNA analysis using the original criteria (|Log_2_FC|>5 and FDR < 0.001). Finally, mSC markers and nmSC markers from the SNAT scRNA-seq dataset were compared to the mSC DEGs identified from the SNAT myelination bulk RNA-seq dataset. We use these comparisons to evaluate the consistency of genes across modalities, species, myelination status, and analysis techniques to identify high-confidence targets for the study of SC differentiation.

### Quantitative Real-time PCR (qRT-PCR)

Total RNA for was extracted using a Quick-RNA Miniprep Plus Kit (Zymo Research #R1058). RNA concentration was measured using a NanoDrop 1000. A total 0.5–2 μg of RNA was used to generate cDNA using a Thermo Scientific^™^ Verso cDNA Synthesis Kit (#AB1453B). qRT-PCR was performed using Applied Biosystems^™^ PowerUp^™^ SYBR^™^ Green Master Mix (#A25742). Primer sequences used in this study are listed in Table S8.

### CRISPR/Cas9 Mediated Deletion of Merlin (NF2)

To genetically disrupt Merlin (*NF2*) in the 101.J iPSC line, single gRNAs were designed (gRNA# AACCCAAGACGTTCACCGTG AGG) to edit the exon 1 region of the *NF2* gene. The Cas9-GFP protein (IDT #10008100) was used during nucleofection with gRNA. The gRNA was designed according to gRNA IVT synthesis kit design and proceeded according to the manual protocol. RNP complex (3–4 μM per reaction) was prepared using a ratio of 1:1.2 Cas9 to sgRNA. Cells were nucleofected with RNP complex in a Lonza nucleofector. After nucleofection, cells were plated into 6 well plates and incubated for 48 hours. Two days after the transfection, 70–80% of cells showed high levels of green fluorescent protein (GFP) expression. After one week, single-cell dilution was performed to isolate individual cells into 96-well plates, targeting a density of one cell per well. Single cell clones were allowed to expand for approximately two to three weeks before being harvested and sent for Sanger sequencing. Merlin protein expression was evaluated by western blot using Merlin antibody (Cell Signalling #6995S). Multiple heterozygous clones were obtained, and then we proceeded with three selected NF2 knockout (KO) clones (#35, #36b, #48) for SCP differentiation, qRT-PCR and western blot analysis.

## RESULTS

### Morphological and immunofluorescence-based validation of hiPSC induced differentiation to SPCs and SCs

After 12 successive passages, the 101.J iPSC line was characterized for the expression of pluripotency markers including SOX2, SSEA4, NANOG, TRA-1–60 and OCT4 by immunostaining. As expected, these pluripotency markers were highly expressed in the101.J iPSC line (**Fig. S1A-C**). The plasticity of the 101.J iPSC line was further assessed by embryoid body (EB) formation, demonstrating the potential to differentiate into all three germ layers, which was confirmed through the expression of Vimentin (mesoderm), β1 tubulin (ectoderm) and SOX17 (endoderm) (**Fig S1D**).

**Figure 1:**
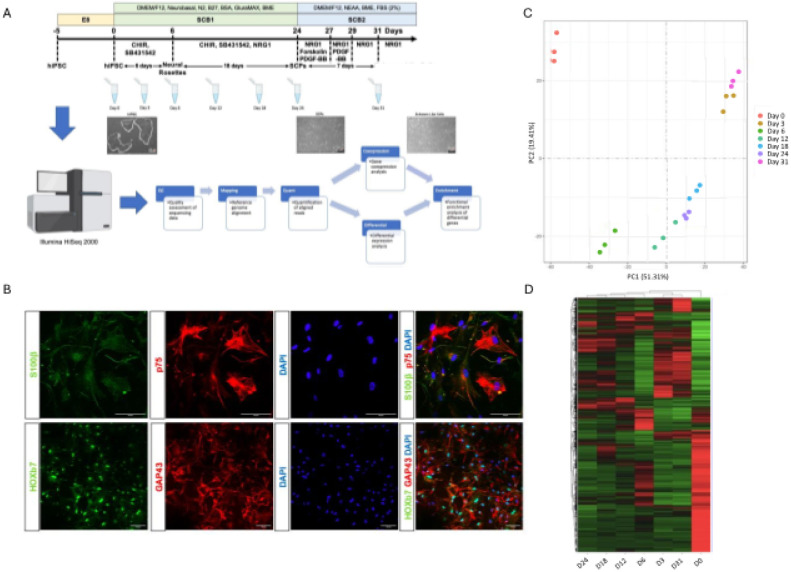
Workflow and validation of directed differentiation of hiPSCs into human SCs including different stages where sample collected for sequencing analysis. **A)** Schematic representation of the differentiation of hiPSCs into SCPs and SCs (Upper panel). Representative bright-field images showing the process of differentiation into SCPs and SCs. Scale bar, 100 mm (middle panel). Steps involve sequencing and data analysis (lower panel). **B)** SC differentiation was confirmed by positive immunocytochemical staining for S100β (green), p75 (red) in upper panel and HOXb7 (green), GAP43 (red) in lower panel after 31 days of differentiation. DAPI (blue) was used to stain the cell nuclei. Scale bars, 100 mm. **C)** Timepoints can be stratified into stages of differentiation by PCA. **D)**Heatmap of differentially expressed genes across different timepoints where the rows (genes) were clustered using hierarchical clustering.

To confirm successful differentiation of hiPSCs into SCPs and subsequently SCs, we performed morphological and IF staining of cells collected at seven timepoints along the hiPSC to SC continuum (Fig. 1A, Table 1). We observed morphological changes during the differentiation of hiPSC to SCPs and SCs where the cells exhibited increasing levels of spatial organization as they became more differentiated (Fig. 1A). SCs are derived from the neural crest and characterized by the expression of distinct makers including S100β, p75, HOXb7, and GAP43, which increased during differentiation into the SC stage (Fig. 1B, Fig. S2). SCP and SC marker gene expression was also validated by qRT-PCR. *SOX17, SOX10, MPZ, NGFR, GAP43, SLUG, S100β, GFAP, CDH19, PMP22*, and *PAX3* were highly expressed in iPSC derived SCPs and SCs but not present in iPSCs. Contrastingly, pluripotency marker genes, *NANOG* and *SOX2*, were highly expressed in iPSCs but significantly reduced in SCPs and SCs (**Fig. S2**). Flow cytometry analysis further confirmed robust SOX10 and GAP43 expression in SCPs, as well as S100β protein expression in SCs (**Fig. S3**). Collectively, these findings indicate that our methodology can efficiently generate SCPs and SCs from hiPSCs with high fidelity.

### Differential gene expression during SC differentiation

To characterize dynamic gene expression changes during SC differentiation, we performed RNA sequencing of cells collected at seven discrete stages along the hiPSC to SC continuum (Table 1). As anticipated, replicates from each stage of differentiation clustered together in principal component analysis (PCA) space (Fig. 1C). Notably, the hiPSC stage (day 0) had clear separation from all other stages (Fig. 1C) and the neural rosette (NR) through SCP stages of differentiation (days 6–24) clustered more closely together compared to other stages (Fig. 1C). There were considerable transcriptional differences between all stages of differentiation, most notably hiPSCs vs later stages of differentiation (Fig. 1D, **Fig. S4-S5**). We identified 18 genes that were significantly up-regulated DEGs in all 6 comparisons (**Table S1**). Of these, only *ANXA1* and *CDH6* were also among the top 5 most significant up-regulated DEGs (lowest FDR adjusted p-values) in any of the timepoint comparisons (Table 2). Notably, CDH6 is associated with cell-cell adhesion and ANXA1 has been implicated in Schwann cell proliferation and migration [47]. Of the 30 total top 5 up-regulated DEGs per timepoint comparison, 18 were unique across all timepoints. These 18 genes were enriched for ontology terms related to cell adhesion (GO:0007155, *P* < 0.001) and anatomical structure formation involved in morphogenesis (GO:0048646, *P* < 0.001). In total, 50 significantly down-regulated DEGs were identified at each timepoint comparison (**Table S1**). However, only four were also found in the top 5 most significant down-regulated DEGs (lowest FDR adjusted p-values) at any of the timepoint comparisons (Table 2). Of the top 5 down-regulated DEGs per timepoint comparison, 18 genes were unique across all timepoints and were enriched for ontology terms related to the regulation of cell fate specification (GO:0042659, *P < 0.001*).

### Gene co-expression modules associated with differentiation, axon guidance and ECM

**Figure 2:**
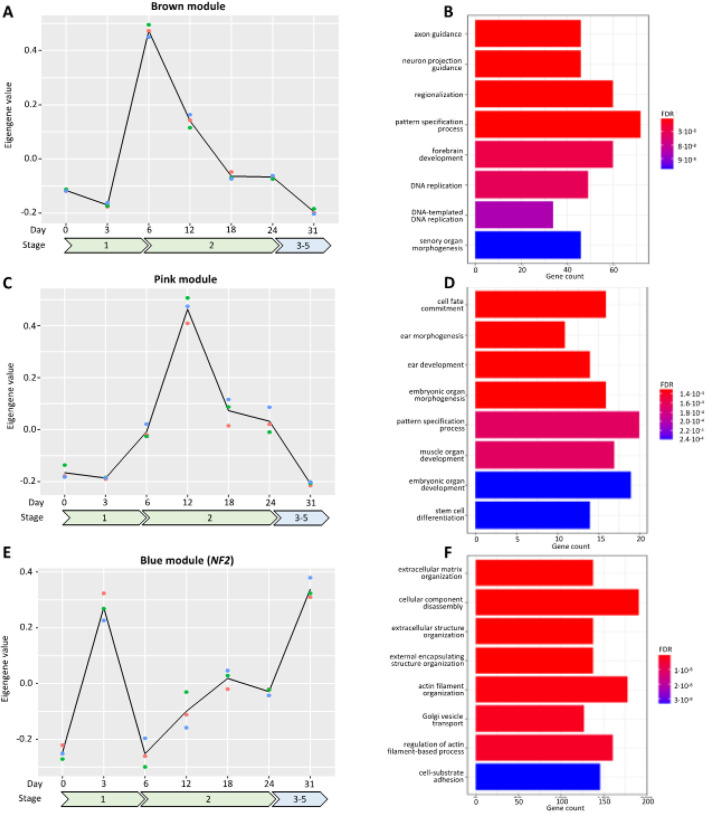
Module eigengene values change over hiPSC differentiation timepoints and are related to tissue structure and neural development. **A)** Brown module eigengene changes over the study timepoints. **B)** Significant ontology terms from functional enrichment analysis for brown module. **C)** Pink module eigengene changes over the study timepoints. **D)** Significant ontology terms from functional enrichment analysis for the pink module. **E**) Blue module (*NF2*) eigengene changes over the study timepoints. **F)** Significant ontology terms from functional enrichment analysis for the blue module (*NF2*).

Building on the results of our differential gene expression analysis, we next performed weighted gene co-expression network analysis (WGCNA) to identify co-regulated gene modules during hiPSC differentiation to SCs. WGCNA identified 24 modules (**Fig. S6**), and 11 of these had significantly altered eigengene values during differentiation (i.e., *P < 0.05*, Table 3). Of the 11 differentiation-associated modules, 7 had significantly enriched ontology terms associated with them (Table 3). The most significant module identified by ANOVA was the brown module (*P < 0.001*, Table 3, Fig. 2A) which had an eigengene peak at day 6, i.e., a significant increase from day 3 to day 6 (*P < 0.001*, Fig. 2A) followed by a decrease from day 6 to day 12 (*P < 0.001*, Fig. 2A). Given the close relationship between SC development and neurons, we were intrigued that the brown module was functionally enriched for axon guidance (P < 0.001, Fig. 2B), forebrain development (*P < 0.001*, Fig. 2B), and sensory organ morphogenesis (*P < 0.001*, Fig. 2B).

The next two most significant modules were red (*P < 0.001*) and turquoise (*P < 0.001*) (Table 3). The red module eigengene steadily increased from hiPSC to SCP stage (*P < 0.001*, i.e., day 0 to day 24, **Fig. S7**) then dramatically decreased from SCP to SC stage (*P < 0.001*, i.e., day 24 to day 31, **Fig. S7**) and was functionally enriched for biosynthetic pathways (**Fig. S7**). In contrast, the turquoise module eigengene, which was functionally enriched for exocytosis (*P < 0.001*, **Fig. S8**) and regulation of neuron projection development (*P < 0.001*, **Fig. S8**) decreased dramatically from the hiPSC to preneural rosette (pre-NR) stage (*P < 0.001*, i.e., day 0 to day 3, **Fig. S8**) and remained suppressed through terminal SC differentiation (*P = 0.732*, i.e., day 3 to day 31, **Fig. S8**). Another pair of modules that showed distinct expression patterns were the pink and blue modules (*P < 0.001* and *P < 0.001* respectively, Table 3). Similar to the brown module that had a distinct eigengene peak at day 6, the pink module peaked at day 12, i.e., a significant increase from day 6 to day 12 (*P < 0.001*, Fig. 2C) and decreased thereafter from day 12 to day 18 (*P < 0.001*, Fig. 2C). This module had numerous ontology terms related to cell fate commitment and ear development (Fig. 2D). The blue module eigengene also had a peak early in hiPSC to SC differentiation at day 3, i.e., a significant increase from day 0 to day 3 (*P < 0.001*, Fig. 2E) and decrease from day 3 to day 6 (*P < 0.001*, Fig. 2E). However, this module eigengene gradually increased again from NR through completion of SC maturation (*P < 0.001*, Fig. 2E). Notably, the blue module contains the NF2 tumor suppressor gene and had significant functional enrichment for extracellular matrix organization (*P < 0.001*, Fig. 2F), extracellular structure organization (*P < 0.001*, Fig. 2F), and cell-substrate adhesion (*P < 0.001*, Fig. 2F). Taken together with the remaining modules (**Fig S9-S14**), the results of our WGCNA reveal that hiPSC differentiation to SCs is associated with dynamic changes in gene expression that are reflective of SC biological function and show clear associations with neural morphology, ear development, and cellular organization.

### Signatures of hAMSC to SC differentiation are unique at the single cell level

**Figure 3:**
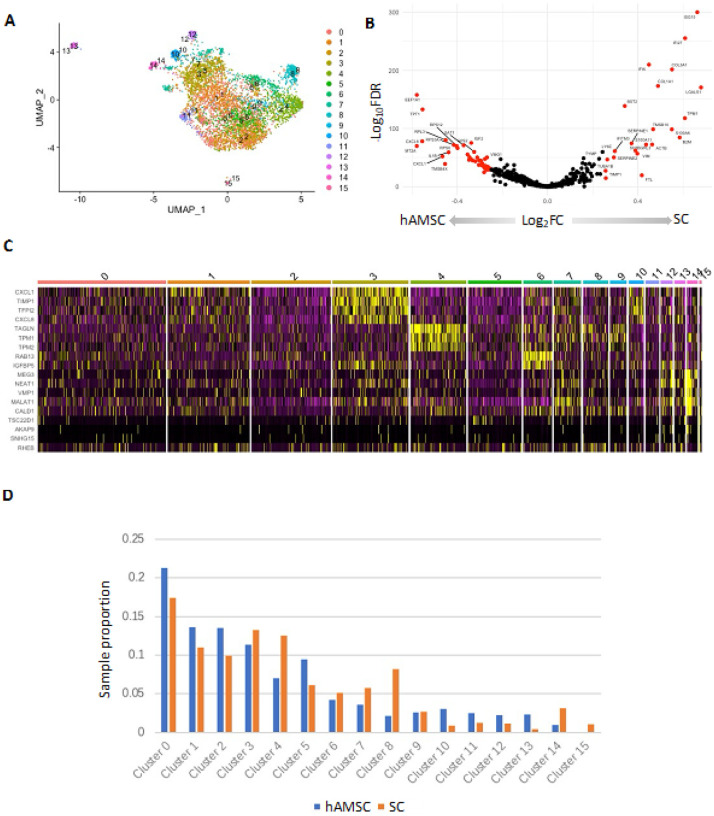
SC-SUMC scRNA-seq analysis. **A)** Scatter plot of 16 clusters identified from the integrated dataset of GSE161066. **B)** Volcano plot of differential expression genes when compared between hAMSCs and SCs. **C)** Heatmap of cluster specific markers. **D)** Proportion of the clusters in both hAMSCs and SCs from the integrated dataset of GSE161066.

To evaluate the robustness and generalizability of our findings, we extended our analysis to examine whether similar gene modules and pathways were retained in other complementary models of human SC differentiation. Wei and colleagues performed single cell RNA-sequencing of SCs derived from human amniotic mesenchymal stem cells (hAMSCs) to identify heterogenous SC populations [29] (GSE161066, Table 1). The SC-SUMC scRNA-seq data contained 3,892 hAMSCs and 1,729 SCs. After QC and dimensionality reduction, we identified a total of 16 clusters (Fig. 3A). DGE analysis revealed clear differences between hAMSC and SCs. Some of the most upregulated genes in SC compared to hAMSCs included ECM related genes including *COL1A1* and *COL3A1* (Fig. 3B, Table 4). To further characterize each cluster, we performed cluster-specific marker analysis using Seurat to identify distinct gene sets highly expressed in each cluster (Fig. 3C). We observed that some clusters had a higher proportion of either hAMSCs or SCs, suggesting that they may represent different stages of differentiation (Fig. 3D). For example, cluster 8 and 14 comprised of a higher proportion of SC sample compared to the hAMSC sample (Fig. 3D) and both of these clusters overexpressed ECM-related markers. Cluster 8 overexpressed *VIM*, and cluster 14 overexpressed *ITGA1, COL1A1*, and *ADAMTS6* (Fig. 3C, **Table S2**).

### Evaluation of SC differentiation in mouse sciatic nerve

To further validate our hiPSC model of SC differentiation *in vitro*, we further compared our transcriptomic data with an *in vivo* dataset comprehensively cataloging SC development in the mouse sciatic nerve (Table 1). Gerber and colleagues generated gene expression profiles from SC isolated from mouse sciatic nerve throughout embryogenesis and postnatal development, which are publicly available through the Sciatic Nerve Atlas, i.e., SNAT, [30]. By integrating bulk and single cell RNA-seq data from SNAT (Table 1), we identified unique and conserved gene signatures of SC differentiation across species and modalities.

**Figure 4:**
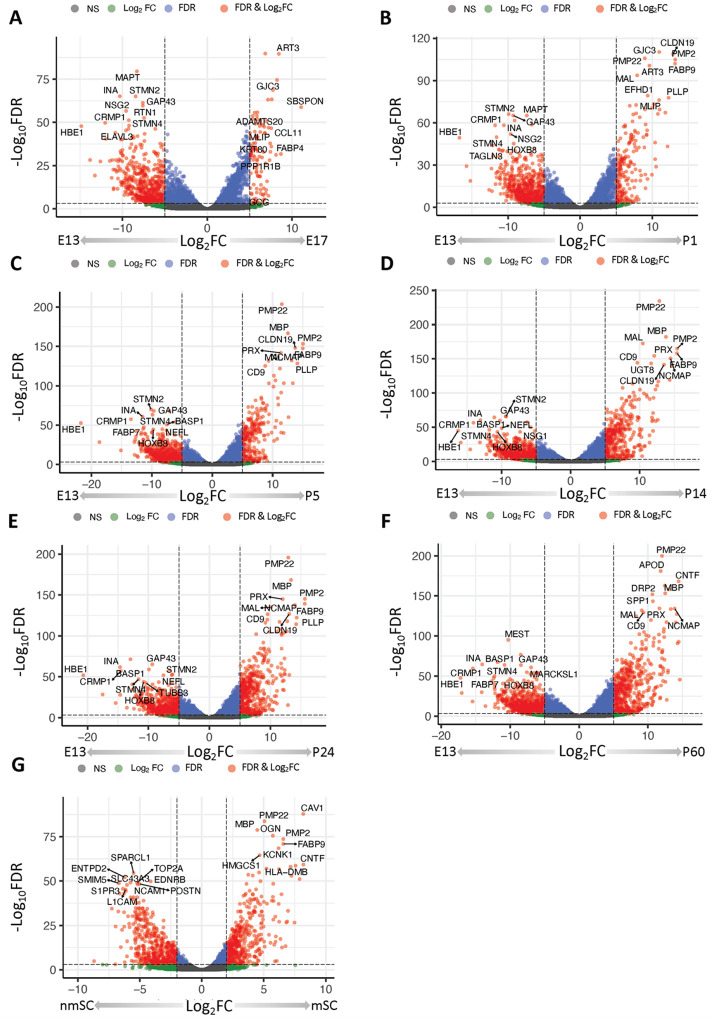
SNAT RNA-seq analysis. DGE results are listed for E13 stage vs. E17 stage **(A)**, E13 stage vs. P1 stage **(B)**, E13 stage vs. P5 stage **(C)**, E13 stage vs. P14 stage **(D)**, E13 stage vs. P24 stage **(E)**, E13 stage vs. P60 stage **(F)**, and mSC/nmSCs **(G)**.

A total of 47 genes were significantly up-regulated at each timepoint comparison (Fig. 4A–F, **Table S1, Table S3**). These genes represented myelination (GO:0042552, *P < 0.001*) and collagen-containing extracellular matrix (GO:0062023, *P < 0.001*). The 362 down-regulated DEGs significant at all comparisons were enriched for neuron development (GO:0048666, *P < 0.001*) and synaptic signaling (GO:0099536, *P < 0.001*). Furthermore, we identified 9 gene co-expression modules that were significantly altered over developmental stage from E13 to P60. (**Table S4, Fig. S15-S24**). Notably some of these modules were also enriched for terms related to neural development like the SNAT-brown module (GO:0010975, Regulation of neuron projection development, *P < 0.001*, **Table S4**).

### Differential gene expression and enrichment in myelinating versus non-myelinating murine Schwann cells

We next sought to investigate the gene expression profiles of mSCs and nmSCs in the SNAT. mSCs had 478 up-regulated DEGs and 471 down-regulated DEGs in comparison to nmSCs (Fig. 4G, **Table S5**). These up-regulated DEGs were enriched for structural constituents of myelin sheath (GO:0019911, *P = 0.039*), myelination (GO:0042552, *P < 0.001*), and sterol biosynthetic processes (GO:0016126, *P < 0.001)*. Down-regulated DEGs were highly enriched for mitotic cell cycle (GO:0000278, *P < 0.001*), cell division (GO:0051301, *P < 0.001*), and positive regulation of cell cycle processes (GO:0090068, *P < 0.001*).

**Figure 5:**
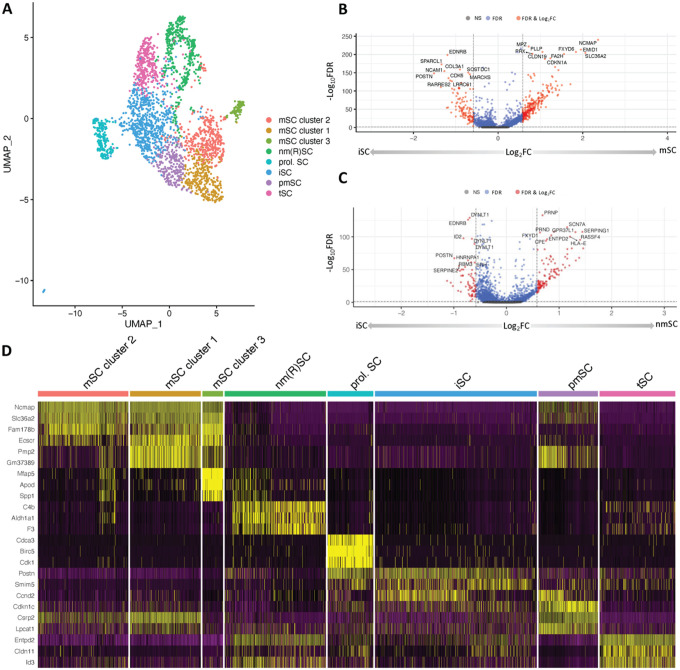
SNAT scRNA-seq analysis. **A)** Scatterplot of 8 clusters identified from the SNAT study representing different stages of Schwann cell development and myelination. **B)** DGE results comparing differentiation from iSC to mSC. **C)** DGE results comparing differentiation from iSC to nmSC. **D)**Heatmap of cluster specific markers (top 3).

The mSC and nmSC subsets remained largely distinct, even upon recomputing the UMAP dimensionality reduction (Fig. 5A)[31]. Two distinct trajectories emerged, both beginning from proliferating SC (prol. SC) and terminating at either mSC cluster 3 or nm(R)SC (Fig. 5A)[31]. We observed 261 up-regulated and 116 down-regulated DEGs in mSCs as compared to iSCs (**Table S6**). As expected, the up-regulated DEGs in mSCs were enriched for terms related to regulation of nervous system development (GO:0051960, *P < 0.001*) and myelination (GO:0042552, *P < 0.001*). However, the most significantly enriched term was sterol biosynthetic process (GO:0016126, *P < 0.001*). The robust upregulation of cell cycle inhibitors like *CDKN1A* (Fig. 5B) in conjunction with the downregulation of cyclins such as *CDK6* (Fig. 5B) highlight the terminal differentiation of this cluster. Using as few as three transcriptomic markers, we could distinguish the stages of SC differentiation and the bifurcation of mSCs and nmSCs (Fig. 5C–D, **Table S7**). Again, we identified ECM-related markers at various differentiation stages including iSCs (*POSTN*), tSCs (*ID3*), and mSCs (*SPP1*) (**Table S7**).

### Differentiating SCs have conserved pathways related to ECM

**Figure 6:**
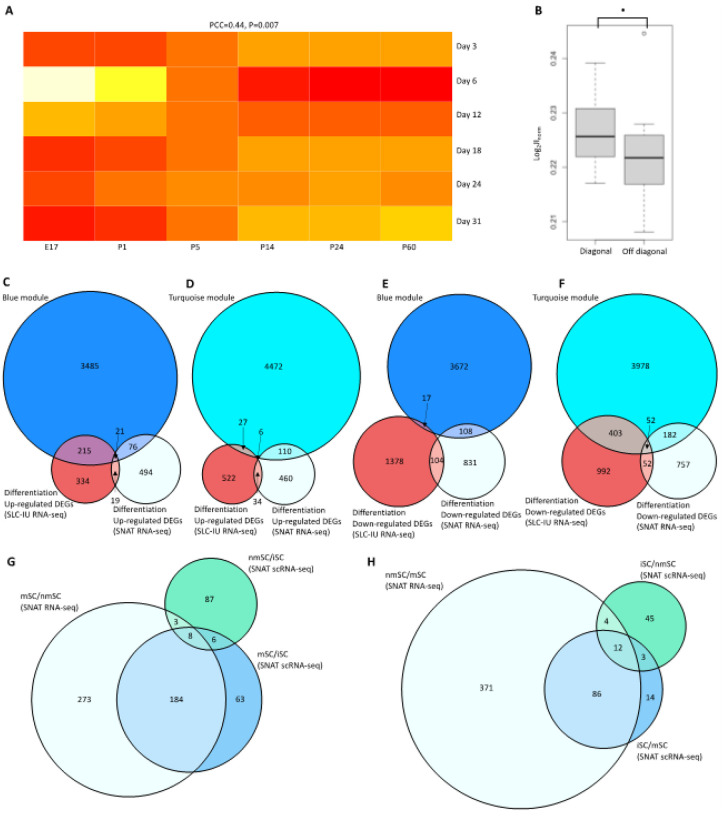
Comparison of identified gene lists. **A)**Normalized JI matrix of averaged SC-IU differentiation up/down-regulated DEGs (|log2FC| > 0.58 and an FDR < 0.05) and SNAT differentiation up/down-regulated DEGs (|log2FC| > 0.58 and an FDR < 0.05) with comparison of JI matrix diagonal to non-diagonal **(B), C)**Comparison of blue module, SC-IU differentiation up-regulated DEGs, and SNAT differentiation up-regulated DEGs, **D)** Comparison of turquoise module, SC-IU differentiation up-regulated DEGs, and SNAT differentiation up-regulated DEGs, **E)** Comparison of blue module, SC-IU differentiation down-regulated DEGs, and SNAT differentiation down-regulated DEGs, **F)** Comparison of turquoise module, SC-IU differentiation down-regulated DEGs, and SNAT differentiation down-regulated DEGs, **G)** Comparison of mSC vs. iSC markers and nmSC vs. iSC markers from SNAT scRNA-seq and mSC up-regulated DEGs from SNAT RNA-seq, **H)** Comparison of iSC vs. mSC markers and iSC vs. nmSC markers from SNAT scRNA-seq and mSC down-regulated DEGs from SNAT RNA-seq. Significance levels are: P>0.10 (n.s.), P<0.10 (·), P<0.05 (*), P<0.01 (**), and P<0.001 (***).

The differentiation processes were not highly conserved between hiPSC and mice. Notably without normalization, there is not a significant correlation between the timepoints in the SNAT dataset and the iPSC differentiation timepoints (**Fig. S25A-F**). However, after accounting for differences in the row and column DEG counts, a moderate association emerged (PCC = 0.44, *P = 0.007*, Fig. 6A). The one-to-one correspondences in DEGs between timepoints (E17-Day 3, P1-Day 6, P5-Day 12, P14-Day 18, P24-Day 24, and P60-Day 31) were also moderately associated (*P = 0.084*, Fig. 6B).

We focused on the blue module and turquoise modules and compared them to the up- and down-regulated DEGs from the SC-IU and SNAT datasets. The blue module contained *NF2* and the eigengene trended upward as time progressed, whereas the turquoise module trended downward. We found that the up-regulated DEGs from both SC-IU and SNAT had high overlap with the blue module, but low overlap with one another (Fig. 6C). In contrast the SC-IU up-regulated DEGs had lower overlap with the turquoise module, while SNAT up-regulated DEGs had a similar number of DEGs in the turquoise module (Fig. 6D). Both the SC-IU and SNAT down-regulated DEGs had lower overlap with the blue module (Fig. 6E) than with the turquoise module (Fig. 6F). Notably 21 genes were up-regulated in both SC-IU, SNAT, and contained in the blue module (**Table S1**). These genes represented specific molecular functions including extracellular matrix structural constituent (GO:0005201, *P = 0.005*) and S100 protein binding (GO:0044548, *P = 0.008*) as well as biological processes including caveola assembly (GO:0070836*, P = 0.006*) and regulation of transforming growth factor beta receptor signaling pathway (GO:0017015, *P = 0.006*).

Aside from these unique conserved processes during mouse SC differentiation and hiPSC to SC differentiation, mSCs had distinct molecular profiles in comparison to nmSCs that were recapitulated in both RNA-seq and scRNA-seq data modalities. Most notably we clearly see that mSC up-regulated DEGs from SNAT RNA-seq have high overlap with mSC markers form SNAT scRNA-seq (Fig. 6G). Similarly, nmSC down-regulated DEGs from SNAT RNA-seq have high overlap with nmSC markers form SNAT scRNA-seq (Fig. 6H).

### NF2 heterozygous KO alters ECM and oncogene transcription

**Figure 7:**
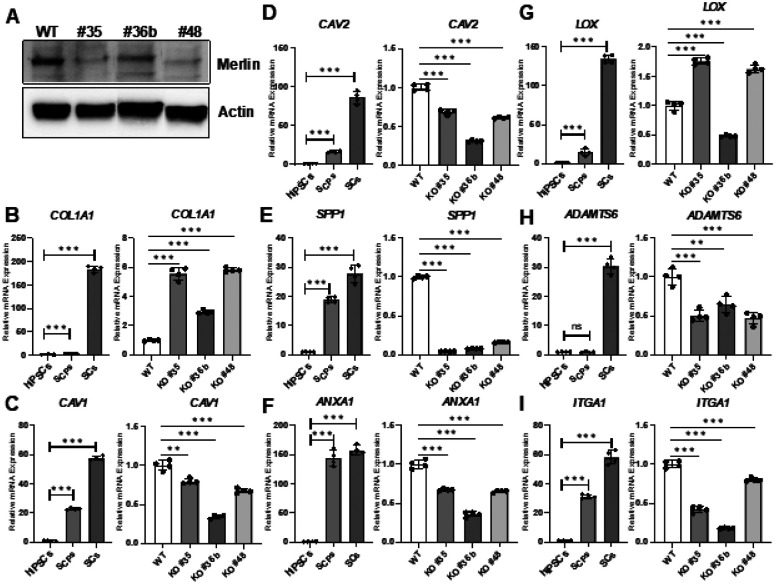
Functional validation of ECM associated markers genes during SC differentiation and following genetic disruption of Merlin (*NF2*). **A)** Western blot of Merlin expression in WT and *NF2* KO hiPSC clones (#35, 36b and 48, **Fig. S29**). The expression of selected ECM associated marker genes including *COL1A1* **(B)**, *CAV1* **(C)**, *CAV2* **(D)**, *SPP1* **(E)**, *ANXA1* **(F)**, *LOX* **(G)**, *ADAMTS6* **(H)**, and *ITGA1* (I) was quantified by qRT-PCR during differentiation from hiPSCs to SCs and in WT and CRISPR-Cas9 edited *NF2* KO clones (#35, 36b and 48). Significance levels are: P>0.10 (n.s.), P<0.10 (·), P<0.05 (*), P<0.01 (**), and P<0.001 (***).

After identifying key regulators of SC differentiation in the *NF2* gene co-expression module, we validated their expression changes using qRT-PCR (**Table S8**) in differentiating SCs and *NF2* heterozygous KO iPSC clones (Fig. 7A, **Fig. S26-S28**). Homozygous KO was not attained due to the effects on cell viability. This analysis revealed alterations in ECM gene expression during SC differentiation and following *NF2* KO. Notably, *COL1A1* expression increased during both differentiation and upon *NF2* KO (Fig. 7B). Conversely, *CAV1, CAV2, SPP1*, *ADAMTS6* and *ITGA1* expression increased during SC differentiation but decreased after *NF2* KO (Fig. 7C–E,H–I). Potential oncogenes, *ANXA1* and *LOX*, both increased in expression during SC differentiation; however, their responses to *NF2* KO differed with *ANXA1* expression decreasing following *NF2* KO, while *LOX* increased (Fig. 7F–G). Collectively, these findings confirm a transcriptional shift in key ECM associated gene during SC differentiation and further implicate a potential role of Merlin (i.e. protein product of *NF2*) in regulating this transcriptional program.

## DISCUSSION

We have developed a state-of-the-art, hiPSC-derived model system to study SC development. We demonstrate that hiPSCs can be efficiently differentiated into SCs based on marker gene expression, IF, flow cytometry analysis and morphological assessment. Furthermore, we identify similar transcriptomic profiles in our hiPSC-SC differentiation model as observed in other external SC differentiation studies [29, 30]. Collectively, this platform will enable further mechanistic study of multiple aspects of SC biology across the differentiation continuum, including but not limited to mSC and nmSC subsets.

Notably, differentially expressed genes during hiPSCs to SC differentiation were significantly associated with DEGs in developing mouse sciatic nerve [30]. However, there were also key differences between our hiPSC model and the mouse model which may be attributable to both species-specific differences as well as the model system employed (*in vitro* vs *in vivo*). Additional studies will also been needed to delineate the impact of hiPSC source (i.e. fibroblasts vs peripheral blood mononuclear cells) on modeling SC development. Indeed, there is already evidence that hiPSC may model the brain better for psychiatric conditions [40] and Alzheimer’s disease [22]. In the case of SC development, this would need to be investigated further.

In addition to validating our model against external datasets, our analysis also revealed novel gene signatures associated with SC development. We identified gene coexpression modules related to differentiation, most notably the blue module that contained the tumor suppressor *NF2*. This module was functionally enriched for multiple terms related to ECM and actin filaments, which is consistent with the relationship between *NF2* and F-actin cytoskeletal defects in schwannoma [41]. The identification of ECM-related genes was found regardless of dataset or analysis technique when analyzing the differentiating SCs. Two blue module genes that are essential to ECM, *CAV1* and *CAV2*, were conserved in both hiPSC and mouse derived SCs and may be linked to *NF2*-associated vesicle trafficking as previously shown in schwannoma [42]. Collagen, *COL1A1* and *COL3A1*, were also overrepresented in our analysis and recently shown to be associated with recurrence of vestibular schwannoma after radiation therapy [43]. Other notable ECM-related genes include that we identified in our analysis with less evidence in schwannoma included: *ITGA1, POSTN, SPP1, ID3, ADAMTS6*. One of the most promising candidates was lysyl oxidase, i.e., LOX, which was present in multiple of our analyses. *LOX* is an essential protein in the ECM that mediates conversion of collagen precursors into reactive forms that can cross-link and stabilize ECM proteins resulting in the regulation of cell adhesion and motility [44]. As a result, *LOX* can play a central role in metastasis [45] and can affect chemotherapy resistance [46].

Growth factors and cell cycle inhibitors, such as *LOX* and *CDKN2B*, were also present in the blue module and conserved in cross species analysis. *LOX* is a tumor suppressor gene, which when mutated in SCs, can suppress T-cells in the melanoma tumor microenvironment [47]. *CDKN2B* is a cell cycle inhibitor that can cause proliferative cell states when deleted or mutated in a multitude of cancer types [48–50]. Furthermore, we identified hiPSC-specific genes of interest that were highly up-regulated during SC differentiation and differentially expressed at all timepoints, namely *ANXA1* and *CDH6*. Finally, *ANXA1* was also identified and is already a promising new target in the SC biology that accelerates SC proliferation and migration [51]. Genetic disruption of Merlin (i.e., protein product of *NF2*) using CRISPR-Cas9 editing in multiple hiPSC derived SCP clones implicates a potential role of Merlin in regulating the transcription of key SC and ECM associated genes.

In summary, we generated a state-of-the-art hiPSC SC differentiation model and performed integrated analyses across five datasets from three independent studies, including both scRNA-seq and RNA-seq. We demonstrate that our hiPSC model robustly recapitulates SC development and identifies novel SC differentiation genes for further study. Furthermore, we identified a strong link in multiple datasets between SC development and ECM that may represent a promising new target in schwannoma. Leveraging this hiPSC platform, furture studies will expand these analyses to include the development of *NF2*-SWN patient derived hiPSCs, generation of hiPSC organoids, and both bulk and single cell multiomic analysis. Future studies using this platform hold promise to reveal new insights into SC biology, *NF2*-SWN, and the role of ECM associated proteins in schwannoma pathogenesis.

## Supplementary Material

Supplementary Files

This is a list of supplementary files associated with this preprint. Click to download.

- SupplementaryMaterials20250607.docx

## Figures and Tables

**Table 1:** Table of the datasets used for analysis in this study.

Dataset name	Samples	Description	Publication	PMID	Accession
SC-IU	21 samples RNA- seq (3 replicates across 7 timepoints) RNA- seq	hiPSC to SC differentiation sampled at seven timepoints Organism: Human	This study	This study	This study
SC-SUMC	2 samples (1 hAMSC sample with 3,892 cells and 1 SC sample with 1,729 cells) scRNA-seq	hAMSC to SC differentiation sampled at hAMSC and SC stages Organism: Human	Wei, Z. frontiers in Physiology. (2021)	34093220	GSE161066
SNAT	28 samples (4 replicates across 7 timepoints) RNA-seq; 8 FACS samples (4 mSC and 4 nmSC) RNA-seq; 8 samples (2 replicates across 4 stages: P1, P5, P14, and P60) scRNA- seq	SCP to SC differentiation; Comparison of mSC and nmSCs; Cellular subtyping of Schwann cells during stages of development. Organism: Mouse	Gerber, D. eLife. (2021)	33890853	GSE137870

**Table 2:** Top DGE analysis results including the top 5 up- and down-regulated DEGs for each of the 6 timepoints (k) after day 0 compared to day 0.

Day k vs. Day 0	Gene	Log2FC	P	FDR
Day 3 Up	*COL8A1*	11.33	0	0
Day 3 Up	*POSTN*	10.15	0	0
Day 3 Up	*SERPINE1*	9.22	0	0
Day 3 Up	*IGFBP3*	7.61	0	0
Day 3 Up	*TGFBI*	7.15	0	0
Day 3 Down	*CD24*	−6.37	5.14E-260	8.69E-257
Day 3 Down	*EPCAM*	−7.93	1.19E-229	1.07E-226
Day 3 Down	*HLA-A*	−5.53	5.65E-213	4.16E-210
Day 3 Down	*PMEL*	−7.55	7.08E-213	5.08E-210
Day 3 Down	*ESRG*	−9.76	7.22E-212	5.06E-209
Day 6 Up	*HAPLN1*	6.97	8.78E-236	2.53E-231
Day 6 Up	*CDH6*	5.91	1.68E-227	2.43E-223
Day 6 Up	*PRTG*	4.91	4.72E-225	4.54E-221
Day 6 Up	*WLS*	5.36	1.57E-184	7.55E-181
Day 6 Up	*GREB1L*	6.90	2.12E-183	8.73E-180
Day 6 Down	*TDGF1*	−5.01	8.98E-198	6.47E-194
Day 6 Down	*ADCY2*	−6.77	1.89E-190	1.09E-186
Day 6 Down	*LINC00678*	−5.15	2.72E-151	7.83E-148
Day 6 Down	*AC064802.1*	−4.65	9.03E-148	2.37E-144
Day 6 Down	*DUSP6*	−4.65	7.95E-145	1.64E-141
Day 12 Up	*ANXA1*	10.95	4.31E-276	6.09E-272
Day 12 Up	*CPE*	5.72	2.35E-191	1.67E-187
Day 12 Up	*PLK2*	5.42	1.95E-190	1.11E-186
Day 12 Up	*PRTG*	4.52	1.72E-185	8.13E-182
Day 12 Up	*CDH6*	5.53	4.22E-181	1.71E-177
Day 12 Down	*TDGF1*	−8.16	0	0
Day 12 Down	*AC064802.1*	−8.52	7.05E-207	6.65E-203
Day 12 Down	*NANOG*	−7.30	7.96E-173	2.82E-169
Day 12 Down	*USP44*	−5.13	5.67E-164	1.46E-160
Day 12 Down	*AC009446.1*	−7.39	2.73E-162	6.42E-159
Day 18 Up	*ANXA1*	10.79	0	0
Day 18 Up	*COL8A1*	10.92	0	0
Day 18 Up	*CAV1*	6.12	0	0
Day 18 Up	*SERPINE1*	8.14	5.48E-295	3.85E-291
Day 18 Up	*POSTN*	8.01	2.79E-294	1.56E-290
Day 18 Down	*TDGF1*	−10.73	2.01E-273	7.06E-270
Day 18 Down	*USP44*	−5.52	1.62E-253	4.54E-250
Day 18 Down	*ZSCAN10*	−6.42	3.59E-216	4.03E-213
Day 18 Down	*ESRG*	−5.16	8.92E-199	7.82E-196
Day 18 Down	*VSNL1*	−7.28	3.70E-179	2.16E-176
Day 24 Up	*ANXA1*	11.19	0	0
Day 24 Up	*POSTN*	8.43	0	0
Day 24 Up	*CAV1*	6.16	0	0
Day 24 Up	*PLK2*	5.77	0	0
Day 24 Up	*SPP1*	4.22	1.63E-291	9.33E-288
Day 24 Down	*USP44*	−5.49	8.04E-274	2.29E-270
Day 24 Down	*ESRG*	−5.53	8.55E-249	1.22E-245
Day 24 Down	*ZSCAN10*	−7.03	1.28E-236	1.52E-233
Day 24 Down	*PMEL*	−4.70	6.92E-211	6.17E-208
Day 24 Down	*B3GNT7*	−6.79	7.86E-201	5.98E-198
Day 31 Up	*CTGF*	6.05	0	0
Day 31 Up	*VIM*	6.55	0	0
Day 31 Up	*COL5A2*	7.04	0	0
Day 31 Up	*COL8A1*	11.75	0	0
Day 31 Up	*POSTN*	9.95	0	0
Day 31 Down	*CD24*	−6.94	0	0
Day 31 Down	*HLA-A*	−6.02	8.07E-296	7.22E-293
Day 31 Down	*SFRP2*	−5.66	3.19E-289	2.61E-286
Day 31 Down	*L1TD1*	−5.78	4.22E-275	3.26E-272
Day 31 Down	*EPCAM*	−7.92	1.74E-267	1.18E-264

**Table 3:** Table of the modules that changed significantly in relation to timepoint evaluated by ANOVA. The number of genes in the module, ANOVA P, and most significant GO term are listed for each module. Modules with no significant GO terms are left blank.

Module	Genes	P	Top GO term	File
Brown	1449	7.66E-17	Axon guidance	File S2
Red	845	2.63E-14	Organic hydroxy compound biosynthetic process	File S3
Turquoise	4615	5.23E-14	Nuclear chromosome segregation	File S4
Cyan	100	1.38E-11		File S5
Pink	692	2.11E-11	Cell fate commitment	File S6
Blue	3797	2.16E-10	Extracellular matrix organization	File S7
Black	833	1.73E-8	Intraciliary transport particle B	File S8
Magenta	159	4.99E-5	Endoplasmic reticulum protein-containing complex	File S9
Yellow	1288	1.52E-2	Cytoplasmic translation	File S10
Midnight Blue	78	4.19E-2	Transcription coregulator activity	File S11
Green Yellow	146	4.26E-2	Methyltransferase activity	File S12

**Table 4:** Top 10 up- and down-regulated DEGs in hAMSCs versus SCs from the SC-SUMC dataset

Gene	Proportion hAMSCs > 0	Proportion SCs>0	Log_2_FC	P	FDR
*ISG15*	0.04	0.44	0.66	1.00E-300	0.00E + 00
*IFI27*	0.02	0.32	0.61	4.98E-256	5.71E-252
*IFI6*	0.01	0.27	0.45	1.37E-210	1.58E-206
*COL3A1*	0.07	0.40	0.55	2.62E-202	3.00E-198
*COL1A1*	0.06	0.34	0.49	6.85E-174	7.86E-170
*LGALS1*	0.49	0.81	0.68	2.36E-171	2.71E-167
*EEF1A1*	0.97	0.88	−0.58	1.66E-158	1.90E-154
*BST2*	0.02	0.22	0.34	1.86E-139	2.14E-135
*TPT1*	0.96	0.88	−0.55	1.79E-133	2.05E-129
*TPM1*	0.31	0.60	0.61	1.85E-118	2.12E-114
*TMSB10*	0.88	0.95	0.47	2.17E-99	2.49E-95
*S100A6*	0.47	0.70	0.55	3.15E-99	3.62E-95
*RPL3*	0.83	0.66	−0.45	8.45E-81	9.70E-77
*CXCL8*	0.71	0.48	−0.55	9.51E-79	1.09E-74
*IGF2*	0.20	0.01	−0.34	6.67E-76	7.65E-72
*RPS3A*	0.80	0.61	−0.42	1.52E-72	1.75E-68
*SAT1*	0.35	0.12	−0.37	7.64E-72	8.77E-68
*MT2A*	0.98	0.98	−0.58	1.37E-70	1.57E-66
*RPS12*	0.89	0.77	−0.40	1.54E-70	1.77E-66
*RPS6*	0.76	0.57	−0.40	6.27E-67	7.20E-63

## Data Availability

The datasets analyzed during the current study are publicly available in the NCBI Gene Expression Omnibus (GEO) repository under the accession numbers GSE161066 and GSE137870. The newly data will be uploaded to GEO upon acceptance of the manuscript.

## Abbreviations

hiPSC: Human induced pluripotent stem cell
SC: Schwann cell
SPC: Schwann cell precursor
iSC: Immature SC
mSC: Myelinating SC
tSC: Transition SC
pmSC: Pro-myelinating SC
nmSC: Not-myelinating SC
nm(R)SC: Non-myelinating (Remak) SC

## References

[1] Fallon M. & Tadi P. Histology, Schwann Cells, in StatPearls (Treasure Island (FL), 2023). 31335036

[2] Salzer J. L. & Zalc B. Myelination Curr. Biol., 26(20): R971–R975. (2016). 27780071 10.1016/j.cub.2016.07.074

[3] Goncalves N. P. Schwann cell interactions with axons and microvessels in diabetic neuropathy. Nat. Rev. Neurol. 13 (3), 135–147 (2017). 28134254 10.1038/nrneurol.2016.201 PMC7391875

[4] Sheikh M. M. & Jesus O. D. Vestibular Schwannoma, in StatPearls. : Treasure Island (FL). (2023).

[5] Carlson M. L. & Link M. J. Vestibular Schwannomas N Engl. J. Med., 384(14): 1335–1348. (2021). 33826821 10.1056/NEJMra2020394

[6] Agnihotri S. The genomic landscape of schwannoma. Nat. Genet. 48 (11), 1339–1348 (2016). 27723760 10.1038/ng.3688

[7] Evans D. G. Neurofibromatosis type 2 (NF2): a clinical and molecular review. Orphanet J. Rare Dis. 4, 16 (2009). 19545378 10.1186/1750-1172-4-16 PMC2708144

[8] Rouleau G. A. Alteration in a new gene encoding a putative membrane-organizing protein causes neuro-fibromatosis type 2. Nature 363 (6429), 515–521 (1993). 8379998 10.1038/363515a0

[9] Evans D. G. A clinical study of type 2 neurofibromatosis. Q. J. Med. 84 (304), 603–618 (1992). 1484939

[10] Leventoux N. Human Astrocytes Model Derived from Induced Pluripotent Stem Cells. Cells, 9(12). (2020). 10.3390/cells9122680 PMC7763297 33322219

[11] Perriot S. Differentiation of functional astrocytes from human-induced pluripotent stem cells in chemically defined media. STAR. Protoc. 2 (4), 100902 (2021). 34746863 10.1016/j.xpro.2021.100902 PMC8551928

[12] Perriot S. Human Induced Pluripotent Stem Cell-Derived Astrocytes Are Differentially Activated by Multiple Sclerosis-Associated Cytokines. Stem Cell. Rep. 11 (5), 1199–1210 (2018). 10.1016/j.stemcr.2018.09.015 PMC6234919 30409508

[13] Voulgaris D., Nikolakopoulou P. & Herland A. Generation of Human iPSC-Derived Astrocytes with a mature star-shaped phenotype for CNS modeling. Stem Cell. Rev. Rep. 18 (7), 2494–2512 (2022). 35488987 10.1007/s12015-022-10376-2 PMC9489586

[14] Abud E. M. iPSC-Derived Human Microglia-like Cells to Study Neurological Diseases. Neuron 94 (2), 278–293e9 (2017). 28426964 10.1016/j.neuron.2017.03.042 PMC5482419

[15] Hasselmann J. & Blurton-Jones M. Human iPSC-derived microglia: A growing toolset to study the brain’s innate immune cells. Glia 68 (4), 721–739 (2020). 31926038 10.1002/glia.23781 PMC7813153

[16] McQuade A. Development and validation of a simplified method to generate human microglia from pluripotent stem cells. Mol. Neurodegener. 13 (1), 67 (2018). 30577865 10.1186/s13024-018-0297-x PMC6303871

[17] Autar K. A functional hiPSC-cortical neuron differentiation and maturation model and its application to neurological disorders. Stem Cell. Rep. 17 (1), 96–109 (2022). 10.1016/j.stemcr.2021.11.009 PMC8758945 34942087

[18] Vierbuchen T. Direct conversion of fibroblasts to functional neurons by defined factors. Nature 463 (7284), 1035–1041 (2010). 20107439 10.1038/nature08797 PMC2829121

[19] Brennand K. J. Modelling schizophrenia using human induced pluripotent stem cells. Nature 473 (7346), 221–225 (2011). 21490598 10.1038/nature09915 PMC3392969

[20] Page S. C. Electrophysiological measures from human iPSC-derived neurons are associated with schizophrenia clinical status and predict individual cognitive performance. Proc. Natl. Acad. Sci. U S A, 119(3). (2022). 10.1073/pnas.2109395119 PMC8784142 35017298

[21] Park I. H. Disease-specific induced pluripotent stem cells. Cell 134 (5), 877–886 (2008). 18691744 10.1016/j.cell.2008.07.041 PMC2633781

[22] Penney J., Ralvenius W. T. & Tsai L. H. Modeling Alzheimer’s disease with iPSC-derived brain cells. Mol. Psychiatry. 25 (1), 148–167 (2020). 31391546 10.1038/s41380-019-0468-3 PMC6906186

[23] Krieger T. G. Modeling glioblastoma invasion using human brain organoids and single-cell transcriptomics. Neuro Oncol. 22 (8), 1138–1149 (2020). 32297954 10.1093/neuonc/noaa091 PMC7594554

[24] Sancho-Martinez I. Establishment of human iPSC-based models for the study and targeting of glioma initiating cells. Nat. Commun. 7, 10743 (2016). 26899176 10.1038/ncomms10743 PMC4764898

[25] Schloo C. & Kutscher L. M. Modeling brain and neural crest neoplasms with human pluripotent stem cells. Neuro Oncol. 25 (7), 1225–1235 (2023). 36757217 10.1093/neuonc/noad034 PMC10326493

[26] Zhang S. Development of human induced pluripotent stem cell (iPSC) line from a 60year old female patient with multiple schwannoma. Stem Cell. Res. 19, 31–33 (2017). 10.1016/j.scr.2016.12.022 28413001

[27] Langfelder P. & Horvath S. WGCNA: an R package for weighted correlation network analysis. BMC Bioinform. 9, 559 (2008). 10.1186/1471-2105-9-559 PMC2631488 19114008

[28] Garcia-Moreno A. Functional Enrichment Analysis of Regulatory Elements. Biomedicines, 10(3). (2022). 10.3390/biomedicines10030590 PMC8945021 35327392

[29] Wei Z. A Subpopulation of Schwann Cell-Like Cells With Nerve Regeneration Signatures Is Identified Through Single-Cell RNA Sequencing. Front. Physiol. 12, 637924 (2021). 34093220 10.3389/fphys.2021.637924 PMC8171402

[30] Gerber D. Transcriptional profiling of mouse peripheral nerves to the single-cell level to build a sciatic nerve ATlas (SNAT). Elife, 10. (2021). 10.7554/eLife.58591 PMC8064760 33890853

[31] Kim H. S. Schwann Cell Precursors from Human Pluripotent Stem Cells as a Potential Therapeutic Target for Myelin Repair. Stem Cell. Rep. 8 (6), 1714–1726 (2017). 10.1016/j.stemcr.2017.04.011 PMC5469943 28506533

[32] Liu Z. Specific marker expression and cell state of Schwann cells during culture in vitro. PLoS One. 10 (4), e0123278 (2015). 25859851 10.1371/journal.pone.0123278 PMC4393255

[33] Chen J. ToppGene Suite for gene list enrichment analysis and candidate gene prioritization. Nucleic Acids Res., (2009). 37(Web Server issue): pp. W305–W311. 19465376 10.1093/nar/gkp427 PMC2703978

[34] Hao Y. Integrated analysis of multimodal single-cell data. Cell 184 (13), 3573–3587e29 (2021). 34062119 10.1016/j.cell.2021.04.048 PMC8238499

[35] Choudhary S. & Satija R. Comparison and evaluation of statistical error models for scRNA-seq. Genome Biol. 23 (1), 27 (2022). 35042561 10.1186/s13059-021-02584-9 PMC8764781

[36] Lause J., Berens P. & Kobak D. Analytic Pearson residuals for normalization of single-cell RNA-seq UMI data. Genome Biol. 22 (1), 258 (2021). 34488842 10.1186/s13059-021-02451-7 PMC8419999

[37] Korsunsky I. Fast, sensitive and accurate integration of single-cell data with Harmony. Nat. Methods. 16 (12), 1289–1296 (2019). 31740819 10.1038/s41592-019-0619-0 PMC6884693

[38] Tran H. T. N. A benchmark of batch-effect correction methods for single-cell RNA sequencing data. Genome Biol. 21 (1), 12 (2020). 31948481 10.1186/s13059-019-1850-9 PMC6964114

[39] McInnes L., Healy J. & Melville J. Umap: Uniform manifold approximation and projection for dimension reduction. arXiv preprint arXiv:1802.03426, (2018).

[40] Deng B. Mouse models and induced pluripotent stem cells in researching psychiatric disorders. Stem Cell. Investig. 4, 62 (2017). 10.21037/sci.2017.06.10 PMC5539390 28815173

[41] Bashour A. M. The neurofibromatosis type 2 gene product, merlin, reverses the F-actin cytoskeletal defects in primary human Schwannoma cells. Mol. Cell. Biol. 22 (4), 1150–1157 (2002). 11809806 10.1128/MCB.22.4.1150-1157.2002 PMC134629

[42] Hennigan R. F. The NF2 tumor suppressor regulates microtubule-based vesicle trafficking via a novel Rac, MLK and p38(SAPK) pathway. Oncogene 32 (9), 1135–1143 (2013). 22525268 10.1038/onc.2012.135 PMC4260777

[43] Shi Q. Collagen Family Genes Associated with Risk of Recurrence after Radiation Therapy for Vestibular Schwannoma and Pan-Cancer Analysis. Dis Markers, 2021: p. 7897994. (2021). 34691289 10.1155/2021/7897994 PMC8528601

[44] Kagan H. M. & Li W. Lysyl oxidase: properties, specificity, and biological roles inside and outside of the cell. J. Cell. Biochem. 88 (4), 660–672 (2003). 12577300 10.1002/jcb.10413

[45] Rachman-Tzemah C. Blocking Surgically Induced Lysyl Oxidase Activity Reduces the Risk of Lung Metastases. Cell. Rep. 19 (4), 774–784 (2017). 28445728 10.1016/j.celrep.2017.04.005 PMC5413586

[46] Saatci O. Targeting lysyl oxidase (LOX) overcomes chemotherapy resistance in triple negative breast cancer. Nat. Commun. 11 (1), 2416 (2020). 32415208 10.1038/s41467-020-16199-4 PMC7229173

[47] Kruglov O. Melanoma-associated repair-like Schwann cells suppress anti-tumor T-cells via 12/15-LOX/COX2-associated eicosanoid production. Oncoimmunology 12 (1), 2192098 (2023). 36998620 10.1080/2162402X.2023.2192098 PMC10044150

[48] Chen X., Yu X. & Shen E. Overexpression of CDKN2B is involved in poor gastric cancer prognosis. J. Cell. Biochem. 120 (12), 19825–19831 (2019). 31297846 10.1002/jcb.29287

[49] Tu Q. CDKN2B deletion is essential for pancreatic cancer development instead of unmeaningful co-deletion due to juxtaposition to CDKN2A. Oncogene 37 (1), 128–138 (2018). 28892048 10.1038/onc.2017.316 PMC5759028

[50] Wrensch M. Variants in the CDKN2B and RTEL1 regions are associated with high-grade glioma susceptibility. Nat. Genet. 41 (8), 905–908 (2009). 19578366 10.1038/ng.408 PMC2923561

[51] Xia W. ANXA1 directs Schwann cells proliferation and migration to accelerate nerve regeneration through the FPR2/AMPK pathway. FASEB J. 34 (10), 13993–14005 (2020). 32856352 10.1096/fj.202000726RRR

